# Factors associated with influenza vaccination status of residents of a rural community in Japan

**DOI:** 10.1186/1471-2458-11-149

**Published:** 2011-03-04

**Authors:** Daisuke Matsui, Masako Shigeta, Kotaro Ozasa, Nagato Kuriyama, Isao Watanabe, Yoshiyuki Watanabe

**Affiliations:** 1 Epidemiology for Community Health & Medicine, Graduate school of, Medical Science, Kyoto Prefectural University of Medicine, Kyoto, Japan; 2 Department of Epidemiology Radiation Effects Research Foundation, Hiroshima, Japan

## Abstract

**Background:**

The rate of influenza vaccination in Japan has declined over the past several decades. It is essential to identify community-specific factors that affect attitudes toward vaccination, but such parameters have not yet been fully determined in Japan. The present study used the Health Belief Model (HBM) to identify perceptions of influenza vaccination in a rural Japanese community.

**Methods:**

All subjects were residents of a rural town in the southern part of Kyoto, Japan. An anonymous self-administered questionnaire was mailed to 846 randomly chosen households (containing 2,665 subjects). The survey explored gender, age, history of influenza, and factors associated with obtaining influenza vaccination, based on the HBM.

**Results:**

A total of 1,182 valid responses (response rate, 44.4%) were received. Sources of information that were associated with vaccination decisions were medical facilities for children (OR = 4.21; 95% CI: 1.17-15.1), workplaces for adults (OR = 2.40; 95% CI: 1.22-4.75), medical facilities, town office and family for elderly subjects (OR = 6.18; 95% CI: 2.42-15.7, OR = 5.59; 95% CI: 2.26-13.8 and OR = 3.29; 95%CI: 1.01-10.6). Subjects, in all age groups, who strongly agreed that the vaccine was effective were significantly more likely to be vaccinated (OR = 10.5; 95%CI: 2.68-41.7 for children; OR = 8.85; 95%CI: 4.61-16.9 for adults; OR = 19.9; 95%CI: 8.28-48.0 for the elderly). The vaccination rate of elderly subjects who expressed concerns regarding adverse vaccine effects (OR = 0.34, 95% CI: 0.15-0.78) or who were worried about practical barriers to the vaccination process (OR = 0.13; 95% CI: 0.05-0.31) was significantly lower than in other populations.

**Conclusions:**

Our results indicate that vaccination coverage can be increased if accurate information on personal risk, severity of influenza illness, and efficacy of vaccination are provided by responsible information sources that are easily accessible. Such sources include medical facilities and municipal offices. In addition, barriers and inconveniences associated with vaccination should be removed, especially if they impact on elderly people.

## Background

The World Health Organization (WHO) recommended strategies for reducing the morbidity and mortality associated with annual influenza epidemics in the recent document entitled *Global Agenda on Influenza Surveillance and Control *[1]. The four main strategies of the agenda are ***(i) ***to strengthen disease and virological surveillance both nationally and internationally; ***(ii) ***to increase public knowledge of the health and economic burden of influenza; ***(iii) ***to raise influenza vaccine usage; and, ***(iv) ***to accelerate national and international action on pandemic preparedness.

In Japan, community-based vaccination of schoolchildren to prevent seasonal influenza has been conducted since 1976 as dictated by the country's Vaccination Law. A report on the vaccination status of Japanese schoolchildren has indicated a steep decline in coverage, from about 80% in the late 1970 s to 18% in 1992 [2], because of widespread public concerns about possible adverse effects of the vaccine and/or lack of vaccine effectiveness. In 1994, the amended Vaccine Law changed vaccination practice from "mandatory" to "recommended". Thereafter, influenza vaccination coverage in Japan continued to decrease, even as worldwide vaccine use improved. The level of influenza vaccination in Japan is currently estimated as less than 10 per 1,000 subjects, one of the worst rates of all developed countries [3-5].

A 2001 study analyzed monthly death rates from all causes, and death attributed to pneumonia and influenza, in Japan since 1962, and reported that as the proportion of vaccinated Japanese schoolchildren declined, the influenza-related mortality rate increased [6]. Other studies have reported on the effectiveness of the vaccine in preventing influenza among institutionalized elderly individuals in Japan [7,8]. Based on these studies, and on an analysis of worldwide influenza epidemiology, the Ministry of Health, Labor, and Welfare of Japan stipulated that influenza was a Category II Disease, as defined in the Vaccination Law. The primary goals of treatment of a Category II Disease are prevention of individual infection, to inhibit the spread of infection by reducing disease prevalence, and to reduce the numbers of severely ill patients. In 2001, the Ministry also recommended that individuals aged 65 years or older should be vaccinated, as should those aged 60 years or older who suffer from chronic disease (*i.e.; *a cardiovascular, pulmonary, or renal condition; or HIV infection) [9].

A 2008 study in Japan indicated that vaccine coverage of targeted individuals had risen from 28% in the 2001/2002 season to 52% in 2005/2006 season [9]. The coverage seemed to be still lower than those in other developed countries, for example, a study in six European countries reported that 62.2% of subjects aged 65 years or older were vaccinated [10]. In addition, the subjects of the cited survey in Japan did not include healthcare providers or those under the age of 65 years with chronic diseases; these groups are commonly included in similar surveys performed in other developed countries [4]. This indicates that influenza vaccination coverage in Japan remains below world standards.

The Health Belief Model (HBM) has been widely utilized to study structural factors associated with attitudes and behaviors related to health and welfare [11]. This model is based on a review by Rosenstock et al. that analyzed 40 reports on factors associated with decisions to obtain vaccination during a pandemic of poliomyelitis during the 1950 s in the United States [12]. A modification of this model, promulgated by Becker et al. [13], has been widely applied in diverse fields of healthcare. The HBM contains several primary concepts that seek to predict why subjects take action to prevent an illness, including perceptions on susceptibility, illness severity, benefits of the planned action, barriers to risk-reduction behavior, cues initiating action, and self-efficacy [11].

Many studies have used the HBM to study influenza vaccination over the past 50 years, yet such work has not been performed in Japan. In the present study, we employed an anonymous, self-administered questionnaire, completed by subjects in a rural Japanese population, to evaluate associations between various HBM factors and attitudes toward influenza vaccination [additional file 1]. Based on our results, we suggest strategies that should improve influenza vaccination coverage in Japan.

## Methods

The survey was conducted in a rural town approximately 35 km south of Kyoto City, Japan. The population was 4,998 at the time of taking of the National Census in 2005, of whom 10.1% were aged 15 years or younger, 14.6% were 16 to 29 years, 19.9% were 30 to 49 years, 26.2% were 50 to 64 years, and 29.2% were 65 years or older. A total of 846 households containing 2,665 subjects were randomly selected from town residential records. The survey was sent to 423 households (1,335 subjects) in January 2007 and to 423 different households (1,330 subjects) in 2008. The anonymous, self-administered questionnaire was mailed to all subjects, who were asked to mail back completed forms. In the case of children under 18 years of age, each questionnaire was answered by parents or carers. The purpose of the survey and the anonymous nature of the work were explained in accompanying written documentation.

The survey included questions on gender, age, and history of influenza. All subjects were asked to select up to three major sources of information that had been used to form their basis of opinion on influenza vaccination. Additional HBM-based questions inquired about ***(i) ***perceived efficacy of vaccination, ***(ii) ***perceived potential adverse effects of vaccination, ***(iii) ***practical barriers/inconveniences to vaccination, ***(iv) ***previous frequency of respiratory infections, ***(v) ***perceived vulnerability to influenza, and ***(vi) ***perceived fear of severe influenza illness. Also, smoking history was investigated. The possible responses to questions i, ii, iii, v, and vi were "strongly agree", "moderately agree", "not sure", "moderately disagree", or "strongly disagree". The possible answers to the question on vulnerability to upper respiratory infection (iv) were "yes", "no", or "not sure". For the question about smoking (vii), the possible answers were: "current smoker", "ex-smoker", or "nonsmoker".

Responses were evaluated by logistic regression analysis in which vaccination was the dependent variable and all of gender, factors associated with HBM, and smoking, were independent variables. Odds ratios (ORs), 95% confidence intervals (CIs), and p values were calculated. If the responses to some questions were insufficient, certain response categories were grouped together. All subjects were classified into groups of less than 18 years of age (children), 18-64 years of age (adults), and 65 years of age or older (elderly people), to account for differences in monetary support systems and other societal factors that differentially affect the elderly, employed workers, and students. In calculating ORs for the major sources of information on influenza vaccination, the reported sources were weighted by the reciprocal of the number of the sources (i.e., if a subject reported three sources in his/her answer, each source was weighted as 1/3 in the calculation) and a multivariate analysis was conducted to adjust the relationship of effects between the sources.

We found no significant differences between the 2007 and 2008 results, and thus pooled the data, adjusted by year of survey. In the 2007 exercise, we grouped household members and evaluated the association between subject vaccination and vaccination of his/her family members, in which singles were excluded from the analysis. All analyses were performed using SAS software (SAS Institute, Cary, NC). This study was approved by the Committee for Ethical Matters in Medical Research of Kyoto Prefectural University of Medicine (Authorization Number E-57).

## Results

Table 1 shows the basic characteristics of the study population. A total of 1,182 subjects returned questionnaires bearing responses to at least one question (response rate: 44.4%). In 2007, we received responses from 213 households containing 582 subjects, and, in 2008, from 215 households with 600 subjects. The distribution of household members was 19.8% singles, 40.1% couples, 12.7% families of three, 11.8% families of four, and 15.6% families of five or more, in the 2007 survey. The response rate of elderly subjects was over 50% when the results of both surveys were combined, but the rate was lower for high-school students and adults under 30 years of age.

**Table 1:** Characteristics of subjects who were mailed questionnaires and of enrolled subjects.

	Mailed	Responded				
			
Age group	Total	Total	Males	Females	Unknown	Response rate
Under 1 year	11	5	4	1	0	45%
1 year	16	9	5	4	0	56%
2 years	9	3	3	0	0	33%
Preschool	60	24	12	12	0	40%
Elementary school	102	39	20	19	0	38%
Junior-high school	75	37	14	21	2	49%
High school	70	26	13	13	0	37%
Unknown but <18 years		1	0	1	0	

18 to 29 years	363	110	55	55	0	30%
30 to 49 years	537	229	90	137	2	43%
50 to 64 years	693	304	139	162	3	44%
65 to 79 years	532	275	135	138	2	52%
80 years or older	197	100	39	58	3	51%
Unknown but ≥18 years		20	5	4	11	

Total	2665	1182	534	625	23	44%

Table 2 shows the vaccination rate within each age group. This was significantly higher among the elderly than in other age groups. The difference in vaccination rates between males and females was small for children and elderly subjects, but was higher for female than for young and middle-aged male adults. In Table 2 we already excluded 20 subjects who missed responses about vaccination.

**Table 2:** Vaccination coverage in enrolled subjects.

	Vaccinated	Unvaccinated	Total	Vaccination coverage		
				
Age group	Total (Males, Females)	Total		Total	Males	Females
0 to 2 years	3 (3, 0)	14	17	18%	25%	0%
Preschool	13 (6, 7)	11	24	54%	50%	58%
Elementary school	18 (11, 7)	21	39	46%	55%	37%
Junior-high school	17 (4, 12)	18	35	49%	31%	57%
High school	6 (3, 3)	20	26	23%	23%	23%
Unknown but <18 years	1 (0, 1)	0	1			

18 to 29 years	26 (11, 15)	83	109	24%	20%	27%
30 to 49 years	62 (18, 44)	166	228	27%	20%	32%
50 to 64 years	102 (34, 68)	200	302	34%	24%	43%
65 to 79 years	200 (91, 107)	73	273	73%	68%	78%
80 years or older	76 (32, 43)	22	98	78%	86%	74%
Unknown but ≥18 years	3 (1, 1)	7	10			

Total	527	635	1162	45%	41%	50%

Note: The sum of males and females may not be equal to the total because of missing response to the question about sex.

Table 3 shows the association between information sources and influenza vaccination among 1,141 subjects who indicated both sex and age group. The information sources most significantly associated with the decision to obtain influenza vaccination were: (a) medical facilities for children (OR = 4.21, p = 0.027), (b) workplaces for adults (OR = 2.40, p = 0.011), and (c) medical facilities, the town office and family for elderly people (OR = 6.18, p < 0.001, OR = 5.59, p < 0.001 and OR = 3.29, p = 0.046, respectively). TV/radio was the next most commonly utilized information source for children and adults, and for one-third of the elderly. However, adults who obtained some information on influenza vaccination from TV/radio were significantly less likely to be vaccinated (OR = 0.43, p = 0.025) and elderly people who obtained information from newspapers/magazines were also less likely to be vaccinated (OR = 0.33, p = 0.009). In responses to the question, only one source was chosen by 38 to 39% of the subjects in all age groups, two sources by 17 to 20%, three sources by 34 to 40%. Five subjects chose four sources and were included in the analysis in the same manner, and the rest did not answer (i.e., missing). Most common combinations of two sources in their answers were the combination of 1) newspapers/magazines and 2) TV/radio that was chosen by 20 to 25% of the subjects in all age groups and that of 2) TV/radio and 4) medical facilities by 10 to 16% of them. Other combinations that were chosen by 10% of the subjects or more were those of 2) TV/radio and 8) family (10% in children), 1) newspapers/magazines and 4) medical facilities, 1) and 5) town office, 2) and 5), and 4) and 5) (13 to 18% in elderly people). There were no combinations of three sources that were chosen by 10% of the subjects or more in any age group.

**Table 3:** Sources for information about influenza vaccination.

Age group	< 18 years (n = 141)				18 to 64 years (n = 634)				≥65 years (n = 366)			
**Information sources**	Vac*	**Unvac**	**OR (95%CI)**	**p**	**Vac**	**Unvac**	**OR (95%CI)**	**p**	**Vac**	**Unvac**	**OR (95%CI)**	**p**

1. Newspapers/magazines	13	26	0.59 (0.14, 2.44)	0.47	52	157	0.55 (0.30, 1.02)	0.058	87	48	0.33 (0.15, 0.76)	0.009
2. TV/radio	22	37	1.27 (0.39, 1.13)	0.68	72	219	0.43 (0.25, 0.74)	0.025	105	48	0.81 (0.36, 1.83)	0.61
3. Internet	1	3	0.62 (0.01, 33.6)	0.81	5	15	1.02 (0.16, 6.40)	0.97	0	0	-	-
4. Medical facilities	22	15	4.21 (1.17, 15.1)	0.027	68	128	1.28 (0.74, 2.20)	0.37	135	20	6.18 (2.42, 15.7)	< 0.001
5. Town office	10	8	2.04 (0.35, 11.6)	0.42	36	79	0.98 (0.50, 1.90)	0.95	143	26	5.59 (2.26, 13.8)	< 0.001
6. Public health center	0	0	-	-	2	4	0.77 (0.03, 17.7)	0.87	6	2	1.07 (0.06, 19.1)	0.96
7. School	9	22	0.37 (0.09, 1.53)	0.17	3	12	0.25 (0.02, 2.21)	0.21	1	0	-	-
8. Family	20	22	3.07 (0.95, 9.82)	0.058	43	84	1.04 (0.56, 1.93)	0.88	49	11	3.29 (1.01, 10.6)	0.046
9. Acquaintances/friends	5	12	0.75 (0.15, 3.78)	0.73	19	51	0.62 (0.27, 1.41)	0.25	25	16	0.73 (0.22, 2.36)	0.60
10. Workplaces	0	2	-	-	45	44	2.40 (1.22, 4.75)	0.011	2	0	-	-
11. Almost none	0	7	-	-	1	32	0.04 (0.01, 0.34)	0.003	2	3	0.49 (0.06, 3.59)	0.048

* Vac: Vaccinated, Unvac: Unvaccinated, OR: Odds ratio, CI: Confidence interval.

-: Not available because of no respondents in vaccinated or unvaccinated group.

Note: ORs were calculated using a multivariate analysis adjusting for gender and year of survey, and values for response to information sources were weighted by the reciprocal of the number of sources selected by the respondent.

Table 4 summarizes HBM factors that were associated with obtaining influenza vaccination. The perception that the vaccine was effective was most significantly associated with the decision to be vaccinated. In particular, subjects who "strongly" agreed that the vaccine was effective were significantly more likely to be vaccinated in all age groups (OR = 10.5, p < 0.001 for children; OR = 8.85, p < 0.001 for adults; OR = 19.9, p < 0.001 for the elderly). Moreover, the extent of agreement that the vaccine was effective appeared to be associated with the probability of vaccination.

**Table 4:** Factors regarding the health belief model associated with obtaining influenza vaccination.

Age group	< 18 years (n = 141)				18 to 64 years (n = 634)				≥65 years (n = 366)			
**Factors regarding the health belief model**	**Vac**	**Unvac**	OR* (95%CI)	**P**	**Vac**	**Unvac**	**OR (95%CI)**	**P**	**Vac**	**Unvac**	**OR (95%CI)**	**p**

Think that influenza vaccination (IV) is effective in preventing influenza illness												
1. Strongly agree	26	17	10.5 (2.68, 41.7)	< 0.001	79	86	8.85 (4.61, 16.9)	< 0.001	179	16	19.9 (8.28, 48.0)	< 0.001
2. Moderately agree	29	45	4.53 (1.22, 16.7)	0.024	95	217	4.25 (2.27, 7.94)	< 0.001	72	37	3.36 (1.48, 7.61)	0.004
3-5. Not sure or moderately/strongly disagree	3	20	1.00		13	121	1.00		12	21	1.00	

Think that IV has potential adverse effects												
1-2. Strongly/moderately agree	30	26	1.20 (0.51, 2.82)	0.67	61	102	0.80 (0.52, 1.22)	0.31	43	12	0.34 (0.15, 0.78)	0.011
3. Not sure	1	18	0.04 (0.01, 0.41)	0.006	11	94	0.16 (0.08, 0.32)	< 0.001	18	13	0.13 (0.05, 0.32)	< 0.001
4. Moderately disagree	9	11	0.79 (0.24, 2.52)	0.69	30	67	0.63 (0.37, 1.05)	0.079	33	11	0.28 (0.11, 0.65)	0.003
5. Strongly disagree	18	18	1.00		86	119	1.00		172	17	1.00	

Have practical barriers/inconveniences to obtaining IV at clinics												
1-2. Strongly/moderately agree	18	22	1.31 (0.54, 3.14)	0.54	38	79	0.84 (0.51, 1.38)	0.49	17	15	0.13 (0.05, 0.31)	< 0.001
3. Not sure	3	17	0.27 (0.07, 1.10)	0.068	18	105	0.30 (0.16, 0.54)	< 0.001	26	13	0.24 (0.10, 0.57)	< 0.001
4. Moderately disagree	12	9	2.11 (0.72, 6.19)	0.17	65	110	1.08 (0.70, 1.68)	0.70	81	26	0.38 (0.19, 0.75)	0.006
5. Strongly disagree	18	27	1.00		65	116	1.00		138	17	1.00	

Have often upper respiratory infection												
1. Yes	7	14	0.89 (0.32, 2.50)	0.84	42	64	1.79 (1.13, 2.83)	0.013	63	13	2.02 (1.02, 4.03)	0.044
2. Not sure	30	49	1.83 (0.83, 4.04)	0.13	94	249	0.99 (0.65, 1.49)	0.96	116	49	1.33 (0.77, 2.28)	0.30
3. No	20	19	1.00		48	129	1.00		88	28	1.00	

Feel vulnerable to influenza illness												
1. Strongly agree	15	16	2.99 (1.02, 6.93)	0.045	24	20	5.06 (2.55, 10.0)	< 0.001	40	3	8.22 (2.36, 28.6)	< 0.001
2. Moderately agree	35	36			87	141	2.61 (1.69, 4.02)	< 0.001	116	32	2.13 (1.20, 3.76)	0.009
3. Not sure	6	14	1.51 (0.37, 6.06)	0.55	32	103	1.28 (0.76, 2.16)	0.34	46	19	1.49 (0.76, 2.94)	0.24
4-5. Strongly/moderately disagree	5	17	1.00		42	177	1.00		62	37	1.00	

Be afraid that influenza illness may become severe when infected												
1. Strongly agree	4	13	2.65 (1.01, 6.93)	0.047	20	26	2.86 (1.46, 5.59)	0.002	54	5	6.84 (2.47, 18.8)	< 0.001
2. Moderately agree	28	29			72	132	2.03 (1.31, 3.14)	0.001	121	32	2.38 (1.32, 4.29)	0.004
3. Not sure	19	16	4.25 (1.45, 12.4)	0.008	44	113	1.39 (0.86, 2.25)	0.17	34	19	1.14 (0.56, 2.32)	0.71
4-5. Strongly/moderately disagree	7	25	1.00		46	171	1.00		52	33	1.00	

Had a hard experience with severe influenza illness previously												
1. Yes	21	20	1.73 (0.79, 3.80)	0.16	53	91	1.65 (1.08, 2.52)	0.018	38	7	1.94 (0.82, 4.59)	0.12
2. No	27	44	1.00		87	244	1.00		178	63	1.00	
3. Not sure/Don't know	10	19	0.97 (0.38, 2.46)	0.95	38	106	1.05 (0.68, 1.63)	0.81	50	22	0.81 (0.45, 1.44)	0.47

* Vac: Vaccinated, Unvac: Unvaccinated, OR: Odds ratio, CI: Confidence interval.

Note: ORs were adjusted for gender and year of survey.

Subjects who were "not sure" about potential adverse effects of vaccination were significantly less likely to be vaccinated, in all age groups (OR = 0.04, p = 0.006 for children; OR = 0.16, p < 0.001 for adults; OR = 0.13, p < 0.001 for the elderly). Elderly subjects who "strongly" or "moderately" believed that the vaccine had adverse effects were also less likely to be vaccinated (OR = 0.34, p = 0.011).

Similar tendencies were evident with respect to practical barriers or inconveniences in obtaining vaccination. Adults and elderly subjects who were "not sure" about barriers or inconvenience were significantly less likely to be vaccinated (OR = 0.30, p < 0.001 for adults; OR = 0.24, p < 0.001 for the elderly). In addition, elderly subjects who "strongly" or "moderately" believed that barriers or inconvenience were associated with vaccination were significantly less likely to be vaccinated (OR = 0.13, p < 0.001). Elderly subjects described these barriers/inconveniences as means of transportation to a clinic, physical disability, and the expense of vaccination *per se*.

Adults and elderly subjects who often suffered from upper respiratory tract infections were significantly more likely to be vaccinated (OR = 1.79, p = 0.013 for adults; OR = 2.02, p = 0.044 for the elderly). Children (via their parents) who "strongly/moderately" believed they were vulnerable to influenza were significantly more likely to be vaccinated (OR = 2.99, p = 0.045). Adults and elderly subjects were more likely to be vaccinated the more strongly they believed that they were vulnerable to influenza (adults: OR = 5.06, p < 0.001 for "strongly agree" and OR = 2.61, p < 0.001 for "moderately agree"; elderly: OR = 8.22, p < 0.001 for "strongly agree" and OR = 2.13, p = 0.009 for "moderately agree").

Regarding the parental perception that an influenza infection may become severe was associated with vaccination of children. Children (*via *their parents) who answered "not sure" (OR = 4.25, p = 0.008) or "strongly/moderately agree" (OR = 2.65, p = 0.047) were significantly more likely to be vaccinated. Adults and elderly subjects who answered "strongly agree" or "moderately agree" were also significantly more likely to be vaccinated (OR = 2.86, p = 0.002 and OR = 2.03, p = 0.001 for adults; OR = 6.84, p < 0.001 and OR = 2.38, p = 0.004 for the elderly). Adults who had previous hard experience of a severe influenza illness were significantly more likely to be vaccinated (OR = 1.65, p = 0.018).

In the 2007 survey, subjects whose family member(s) was/were vaccinated were significantly more likely to be vaccinated, for all of children (OR = 26.3, 95% CI: 10.1-68.5, p < 0.001), adults (OR = 5.31, 95% CI: 3.64-7.73, p < 0.001), and the elderly (OR = 3.72, 95% CI: 2.50-5.55, p < 0.001).

Finally, current smokers were significantly less likely to be vaccinated than were nonsmokers (OR = 0.36, 95%CI: 0.22-0.60, p < 0.001 for adults; OR = 0.26, 95%CI: 0.11-0.61, p = 0.001 for the elderly). Ex-smokers tended to be less likely to be vaccinated than nonsmokers (OR = 0.67, 95%CI: 0.40-1.13, p = 0.13 for adults; OR = 0.80, 95%CI: 0.46-1.40, p = 0.44) for the elderly.

## Discussion

The World Health Assembly (WHA) recommendations for administration of influenza vaccines urged member states to establish and implement strategies to increase vaccination coverage of all people at high risk, including the elderly and those with underlying diseases, with the goal of attaining vaccination coverage of at least 50% by 2006 and 75% by 2010 [14]. To accomplish these objectives, it is imperative that both healthcare providers and the general population have accurate information about the influenza vaccine. Surveys of community perception of vaccination based on the HBM can be used to assess current attitudes toward vaccination.

In the Japanese language, the same word is used to describe "cold" (a general upper respiratory tract infection caused by various viruses) and "flu" (respiratory tract infections specifically caused by influenza viruses), and the general public may thus perceive influenza as a type of "cold" [2], leading to diagnostic misclassification [15]. Moreover, patients in Japan are likely to be greatly influenced by family members or close friends when deciding whether to receive influenza vaccination [16]. Reports by the mass media on potential adverse effects, and/or highlighting doubts about the efficacy of the vaccine, may have altered vaccination perceptions [9]. This motivated our present study of community attitudes toward influenza vaccination.

First, our results indicate that age is an important factor influencing vaccination. The vaccination rate was highest in subjects 2-15 years of age and 65 years of age or older. High coverage of the elderly was expected, because the government strongly recommends influenza vaccination for this group, and provides monetary support for vaccination. In 1999, Chapman et al. reported that 18 studies concluded that olderpeople had higher rates of influenza vaccination among 28 studies thatanalyzed for the elderly [17].

Consistent with previous reports [18-20], our results indicate that smokers were less likely to be vaccinated for influenza than were non-smokers. These findings are troubling because smokers have a higher prevalence of respiratory and cardiac disorders, and are at greater risk of exacerbation and/or complications if they suffer from influenza.

Sources of information on vaccination had a significant effect on the probability of vaccination. In children, information from medical facilities had a positive effect on the vaccination rate. This may be because although the health insurance system of Japan covers everyone, additional support is provided for children. Parents may visit physicians more frequently when their children have mild symptoms, compared to the frequency of such visits in other countries. For adults, we found that information from the workplace had a significantly positive effect on vaccination rate. Employers are obviously motivated to prevent workers from developing infections, and dissemination of information *via *the workplace seems to be effective in encouraging working adults to become vaccinated. Recently, some workplaces, especially those of healthcare and human services professionals, have recommended influenza vaccination to workers. Such measures may have influenced the results of this study and may lead to improved vaccination coverage among adults.

We found that elderly individuals who obtained influenza information from medical facilities, town offices, and their family were more likely to be vaccinated. It is probable that retired individuals over 65 years of age are more aware of information provided in town offices than are younger subjects, and the town office appears to efficiently disseminate information to this age group. Previous studies suggested that comments from physicians and/or nursing staff strongly influenced the likelihood of influenza vaccination in elderly patients [17,21-23]. Although family's influence on vaccination was not as strong as previous study in Japan [16], family's opinion is thought to be influential to the elderly since they are often supported to be vaccinated by their family in such a rural town.

In all of our age groups, many subjects obtained information from the mass media, including newspapers, magazines, TV, or radio, but these individuals were less likely to be vaccinated. Although both newspaper/magazine and TV/radio showed a significantly inverse association with vaccination in adults and elderly people in a univariate analysis (data not shown), either one was significant in the multivariate analysis in Table 3 because they were likely chosen together. We suggest that future studies should clarify the roles of mass media and identify methods to improve the provision of responsible data on influenza vaccination to the general public.

Our results also indicate that perception of the efficacy of vaccination was the most significant factor associated with obtaining vaccination. In particular, subjects from all three age groups who "strongly" agreed that vaccination was effective were very likely to be vaccinated. In agreement with our results, previous studies of populations from diverse countries and of various ethnicities indicated that perception of the efficacy of influenza vaccination was one of the most influential factors determining vaccine acceptance [17,21,23-25]. Therefore, we suggest that advocacy activities, including dissemination of accurate scientific information on the efficacy of influenza vaccination, should be increased.

Subjects in all age groups who were "not sure" about the adverse effects of vaccination had lower rates of vaccination. In addition, elderly people who had moderate or serious concerns about adverse effects were less likely to be vaccinated. This suggests that the perception that the influenza vaccine has adverse effects did not necessarily lead to development of a negative attitude toward influenza vaccination, but seemed to indicate the respondents' interest in the vaccine. These findings are somewhat inconsistent with those of previous studies [16,17,21,24].

For the elderly, practical barriers to and inconveniences associated with vaccination clearly led to a reduction in vaccine coverage. The perceived barriers included transportation issues, physical disabilities, and expense, and indicate that many individuals at high risk of influenza do not have easy access to vaccination. In Japan, many rural towns are located in mountainous regions similar to our study area, with a small population of young people and a large proportion of elderly. Thus, our results are applicable to many areas of Japan, where town offices and other support groups also work to reduce barriers for the elderly. Vaccination of family member(s) was clearly related to subject vaccination status although family members played only small roles as an information sources on influenza vaccination. Family members are thought to share the same attitude, either positive or negative, to influenza vaccination, and subjects were unconscious of the opinions of family members. It is to be expected that younger adults accompanying children or elderly subjects, and married couples, would visit the same clinic.

Based on the HBM, the perception of vulnerability to and the possible severity of influenza were critical factors affecting the probability of vaccination; this was especially true of adults and elderly subjects. In such subjects, the consequences of influenza, such as lost work time and possible severe complications including pneumonia, are relatively common. Although influenza is not infrequent in children, severe complications are rare and parents seem to understand this fact. Vulnerability to the common cold was not associated with the decision to obtain influenza vaccination, suggesting that the general public of Japan understands the distinction between flu and the common cold.

Overall, the probability that any of our subjects obtained influenza vaccination was consistently explained by the HBM. The results of the current study will be useful in developing strategies for rural areas. We suggest that future work should examine other geographical regions, such as large urban centers, where factors influencing vaccination decisions may be different.

One limitation of the present study is the low response rate (44.4%). This may have led to a selection bias toward more health-conscious participants. However, among our subjects aged 65 years or over, vaccination coverage was 74.4%, thus comparable to the coverage of 62.2% indicated by governmental records of monetary support provided by the town office. Such governmental data were not available for the other age groups. Another limitation is that our study was performed over 2 consecutive years, which is not ideal if an epidemic infectious disease is to be investigated. Nevertheless, the results did not significantly differ between the 2 years, and we thus analyzed all data together, after adjustment for year of survey. In addition, we found no difference in government strategy, or the nature of media coverage of influenza vaccination, between 2007 and 2008. In addition, we could not take into account for clustering by household since household contacts were not clarified because of anonymous basis of the survey.

Using the HBM, we evaluated factors affecting the probability that residents of a rural community in Japan would choose to obtain influenza vaccination. Our findings indicate the importance of the availability of accurate information on the risk and severity of influenza, and the efficacy of vaccination, from sources that are easily accessible, such as healthcare providers and town offices. To increase vaccination among the elderly, it is critical to remove practical barriers, such as transportation problems.

## Conclusions

Our results indicate that vaccination coverage can be increased if accurate information on personal risk, severity of influenza illness, and efficacy of vaccination, are provided by responsible information sources that are easily accessible. Such sources would include medical facilities and municipal offices. In addition, barriers to and inconveniences associated with vaccination should be removed, especially if these factors impact on elderly people.

## List of abbreviations used

CI: Confidence interval; HBM: Health Belief Model; OR: Odds ratio; TV: television

## Competing interests

The authors declare that they have no competing interests.

## Authors' contributions

DM participated in the design of the study, collected the data, performed the statistical analysis, drafted the manuscript, and served as the principal investigator. MS conceived the study, participated in coordination thereof, and drafted the manuscript. KO took part in the design of the study, collected the data, performed the statistical analysis, and drafted the manuscript. NK helped to draft the manuscript. IW helped to draft the manuscript. YW supervised the data analysis, participated in study coordination, and helped to draft the manuscript. All authors have read and approved of the final manuscript.

## Pre-publication history

The pre-publication history for this paper can be accessed here:

http://www.biomedcentral.com/1471-2458/11/149/prepub

## Supplementary Material

Additional file 1 **Questionnaires for the subjects of this study**. The file contains two questionnaires used for persons who were 18 years of age or older, and under 18 years of age. They contain questions on the following section; gender, age, history of influenza, and factors associated with obtaining influenza vaccination, based on the Health Belief Model. Click here for file
