# Supplementary material for: Factors associated with influenza vaccination status of residents of a rural community in Japan
Source: BMC Public Health. 2011 Mar 4;11:149. doi: 10.1186/1471-2458-11-149 (PMC3060116; doi:10.1186/1471-2458-11-149)
Supplement: Additional file 1 — Questionnaires for the subjects of this study. The file contains two questionnaires used for persons who were 18 years of age or older, and under 18 years of age. They contain questions on the following section; gender, age, history of influenza, and factors associated with obtaining influenza vaccination, based on the Health Belief Model. [file 1471-2458-11-149-S1.DOC]

[Questionnaire used for persons who were 18 years of age or older]

Survey on Influenza Vaccination

　Will you agree to complete this survey? Please circle either 1. or 2. below.

　　　1. Yes → Please answer the questions below and then send this form back.

　　　2. No → Please send this form back now. Please use the enclosed prepaid envelope.

Please circle the numbers applicable to you. Please write freely when you are invited to comment below.

| Gender | 1. Male. 2. Female. |
| --- | --- |
| Age group (years) | 1. 18～29.　2. 30～49.　3. 50～64.　4. 65～79.　5. 80 or older |

Q1．Did you receive influenza vaccination in the interval October to December, 2007?

| 1. Yes (in a clinic in the town).　2. Yes (in a clinic outside the town). 3. No. | |
| --- | --- |
| How many doses? | 1. One dose.　2. Two doses.　3. Not sure. |

Q2．Where did you find out about influenza vaccination? Please select up to THREE of your major sources.

| 1. Newspapers/magazines.　2. TV/radio.　3. Internet.　4. Medical facilities.  5. Town office (including brochure). 6. Public health center (including brochure).  7. School. 8. Family. 9. Acquaintances/friends. 10. Workplace.  11. Other (specifically: ). 12. I found almost no information. |
| --- |

Q3．What do you think about influenza vaccination?

| Do you think that influenza vaccination (IV) is effective in preventing influenza? | 1. Strongly agree. 2. Moderately agree. 　3. Not sure. 4. Moderately disagree. 　　　5. Strongly disagree. |
| --- | --- |
| Do you think that IV has potential adverse effects? | 1. Strongly agree. 2. Moderately agree.　 3. Not sure. 4. Moderately disagree.　　　 5. Strongly disagree. |
| Is there any practical barrier/inconvenience for you to obtaining IV at clinics? | 1. Strongly agree. 2. Moderately agree.　 3. Not sure. 4. Moderately disagree.　　　 5. Strongly disagree. |
| If so, please tell us exactly what the problems are: |  |
| Please write freely if you have any questions or opinions about IV: |  |

Q4. Health Status.

| Would you say that, in general, your health is: | 1. Good. 2. Fair.　3. Poor. |
| --- | --- |
| Do you catch cold easily? | 1. Yes. 2. No. 3. Not sure. |
| Do you smoke? | 1. Yes.　2. Have quit.　3. No. |

Q5. About your family.

| Do you have any family member in your household who goes to a day nursery, a kindergarten, a primary school, a junior-high school, or a high school? | 1. Yes.　 2. No. |
| --- | --- |
| Do you have any family member in your household who would likely suffer severely from influenza if contracted (*i.e.*; an elderly or physically handicapped person, or an infant)? | 1. Yes.　 2. No.  3. Not sure. |

Q6. What do you think about influenza?

| Do you think there is a high likelihood that you could contract influenza? | 1. Strongly agree. 2. Moderately agree.　 3. Not sure. 4. Moderately disagree.　　　 5. Strongly disagree. |
| --- | --- |
| Do you think your influenza would become severe if you contract it? | 1. Strongly agree. 2. Moderately agree.　 3. Not sure. 4. Moderately disagree.　　　 5. Strongly disagree. |
| Have you ever suffered from high fever or severe symptoms when you had influenza? | 1. Yes.　2. No.　3. Not determine.  4. I don’t know (if you don’t know whether you have had influenza or not). |

Thank you very much.

Please send this back to the town office within a week of receipt using the

enclosed return mail envelope.

[Questionnaire used for persons who were under18 years of age]

**Survey on Influenza Vaccination**

This questionnaire is addressed to the parents/guardians of a child aged under 18 years in your family.

Will you agree to complete the survey?

1. Yes　→ Please answer the questions below and send this form back.

　　　2. No　→ Please send this form back now using the enclosed prepaid envelope.

Below, please circle all numbers applicable to you. Please write freely when you are invited to comment below.

| Child’s gender | | 1. Male. 2. Female. | |
| --- | --- | --- | --- |
| Age of child | 1. Under 1 year. 2. 1 to 2 years. 3. 2 to less than 3 years.  4. Between 3 years and pre-school age. 5. Primary school student.  6. Junior high school student. 7. Graduate from junior high school. | | |
| Does your child attend a day nursery or kindergarten  if he/she is of pre-school age? | | | 1. Yes.　 2. No. |

Q1. Did your child receive influenza vaccination in the interval October to December 2007?

| 1. Yes (in a clinic in the town).　2. Yes (in a clinic outside the town). 3. No. | |
| --- | --- |
| How many doses? | 1. One dose.　2. Two doses. 3. Not sure. |

Q2. Where did you (the parent/guardian) find out about influenza vaccination? Please select up to THREE of your major sources.

| 1. Newspapers/magazines.　2. TV/radio.　3. Internet.　4. Medical facilities.  5. Town office (including brochure). 6. Public health center (including brochure).  7. School. 8. Family. 9. Acquaintances/friends. 10. Workplace.  11. Other (specifically: ). 12. I found almost no information. |
| --- |

Q3. What do you (the parent/guardian) think about influenza vaccination for your child?

| Do you think that influenza vaccination (IV) is effective in preventing influenza? | 1. Strongly agree. 2. Moderately agree.  3. Not sure. 4. Moderately disagree.  5. Strongly disagree. |
| --- | --- |
| Do you think that IV has potential adverse effects? | 1. Strongly agree. 2. Moderately agree.  3. Not sure. 4. Moderately disagree.  5. Strongly disagree. |
| Is there any practical barrier/inconvenience for your child to obtaining IV at clinics? | 1. Strongly agree. 2. Moderately agree.　 3. Not sure. 4. Moderately disagree.　　　 5. Strongly disagree. |

| If so, please tell us exactly what the problems are: |  |
| --- | --- |
| Please write freely if you have any questions or opinions about IV: |  |

Q4. Health Status of your child

| Would you say that, in general, your child’s health is: | 1. Good. 2. Fair.　3. Poor. |
| --- | --- |
| Does your child catch cold easily? | 1. Yes. 2. No. 3. Not sure. |

Q5. About your family

| Do you have any family member in your household (apart from the child who is the subject of this survey) who goes to a daycare center, a kindergarten, a primary school, a junior-high school, or a high school? | 1. Yes.　 2. No. |
| --- | --- |
| Do you have any family member in your household (apart from the child who is the subject of this survey) who would likely suffer severely from influenza if contracted (*i.e.;* an elderly or physically handicapped person, or an infant)? | 1. Yes.　 2. No.  3. Not sure. |

Q6. What do you think about influenza?

| Do you think there is a high likelihood that your child could contract influenza? | 1. Strongly agree. 2. Moderately agree.　 3. Not sure. 4. Moderately disagree.　　　 5. Strongly disagree. |
| --- | --- |
| Do you think your child’s influenza would become severe if he/she contracts it? | 1. Strongly agree. 2. Moderately agree.　 3. Not sure. 4. Moderately disagree.　　　 5. Strongly disagree. |
| Has your child ever suffered from high fever or severe symptoms when the child had influenza? | 1. Yes.　2. No.　3. Not determine.  4. I don’t know (if you don’t know whether you have had influenza or not). |

Thank you very much.

Please send this back to the town office within a week of receipt using the

enclosed return mail envelope.
